# Elongation of Mouse Prion Protein Amyloid-Like Fibrils: Effect of Temperature and Denaturant Concentration

**DOI:** 10.1371/journal.pone.0094469

**Published:** 2014-04-18

**Authors:** Katazyna Milto, Ksenija Michailova, Vytautas Smirnovas

**Affiliations:** Department of Biothermodynamics and Drug Design, Vilnius University Institute of Biotechnology, Vilnius, Lithuania; University of Maryland School of Medicine, United States of America

## Abstract

Prion protein is known to have the ability to adopt a pathogenic conformation, which seems to be the basis for protein-only infectivity. The infectivity is based on self-replication of this pathogenic prion structure. One of possible mechanisms for such replication is the elongation of amyloid-like fibrils.

We measured elongation kinetics and thermodynamics of mouse prion amyloid-like fibrils at different guanidine hydrochloride (GuHCl) concentrations. Our data show that both increases in temperature and GuHCl concentration help unfold monomeric protein and thus accelerate elongation. Once the monomers are unfolded, further increases in temperature raise the rate of elongation, whereas the addition of GuHCl decreases it.

We demonstrated a possible way to determine different activation energies of amyloid-like fibril elongation by using folded and unfolded protein molecules. This approach separates thermodynamic data for fibril-assisted monomer unfolding and for refolding and formation of amyloid-like structure.

## Introduction

Prion protein (PrP) plays a big role in a number of lethal neurological diseases, known as transmissible spongiform encephalopaties. These disorders are associated with aggregation of normal cellular prion protein (PrP^C^) into pathogenic beta-sheet-rich prion isoforms (PrP^Sc^). Although the majority of suspected cases of human prion diseases are sporadic [1], prions are mostly known because of their infectivity. The infectious nature of prion diseases is based on the ability of PrP^Sc^ to self-replicate by converting PrP^C^ into same pathogenic isoform. One of possible mechanisms of pathogenic prion structure replication is elongation of amyloid-like fibrils.

Since the discovery of prions, one of the main tasks in the field was to produce infectious PrP conformation *in vitro*, as this would finally prove the hypothesis of protein-only infection [2]. A number of attempts generated amyloid-like structures [3], [4]. Such prion protein fibrils share some properties of PrP^Sc^ (such as beta-sheet-rich secondary structure and ability to self-replicate by addition of native PrP) but have much shorter proteinase K (PK) resistant cores [5], and show slight infectivity only in mice which overexpress PrP^C^ 16 fold [6]. Later studies showed that PK resistance can be extended by annealing at high temperature [7], or by using protein misfolding cyclic amplification (PMCA) [8]. When examined by hydrogen/deuterium (H/D) exchange, annealed fibrils showed only slight differences in deuterium incorporation, when compared to untreated amyloid-like fibrils [9]. Such fibrils induced disease in hamsters only after the second passage, thus they are not directly infective [10]. PMCA-generated recombinant PrP amyloid-like fibrils have an extended region, protected from H/D exchange [8], and can induce disease in hamsters, though not as effectively as PrP^Sc^ [11]. Studies of H/D exchange on brain-derived infective PrP show similar highly packed structure as in case of recombinant PrP amyloid-like fibrils, but in a much longer region (entire 90–230 region versus 160–220 region) [12]. It suggests the possibility that brain-derived PrP^Sc^ may be similar to amyloid-like fibrils, just with a much longer beta-sheet core region. Recently Ma and co-workers reported a protocol for *de novo* generation of infectious prions from recombinant PrP [13]. It has similar infectivity to brain-derived prions, but due to low efficiency of the protocol, the structure of these prions is still unknown.

The structure of recombinant PrP amyloid-like fibrils was extensively studied by a number of techniques all giving similar conclusions – parallel in-register beta-sheet in the C-terminal region (starting from residues 160–170, up to residues 220–225) and a disordered N-terminus [9], [14], [15]. The difference in structure between the native prion protein and the amyloid form requires a conversion of the native alpha-helices into beta-sheets. Usually, amyloid-like fibrils are formed in the presence of moderate denaturant concentration [3], [4], under conditions where the native protein is at least partially unfolded. However *in vivo*, the environment does not help in unfolding of native helices. We tried to test if prion protein amyloid-like fibrils self-replicate using stable native PrP, and how environmental factors (such as temperature and denaturant concentration) affect fibril elongation kinetics.

## Results and Discussion

**Figure 1:**
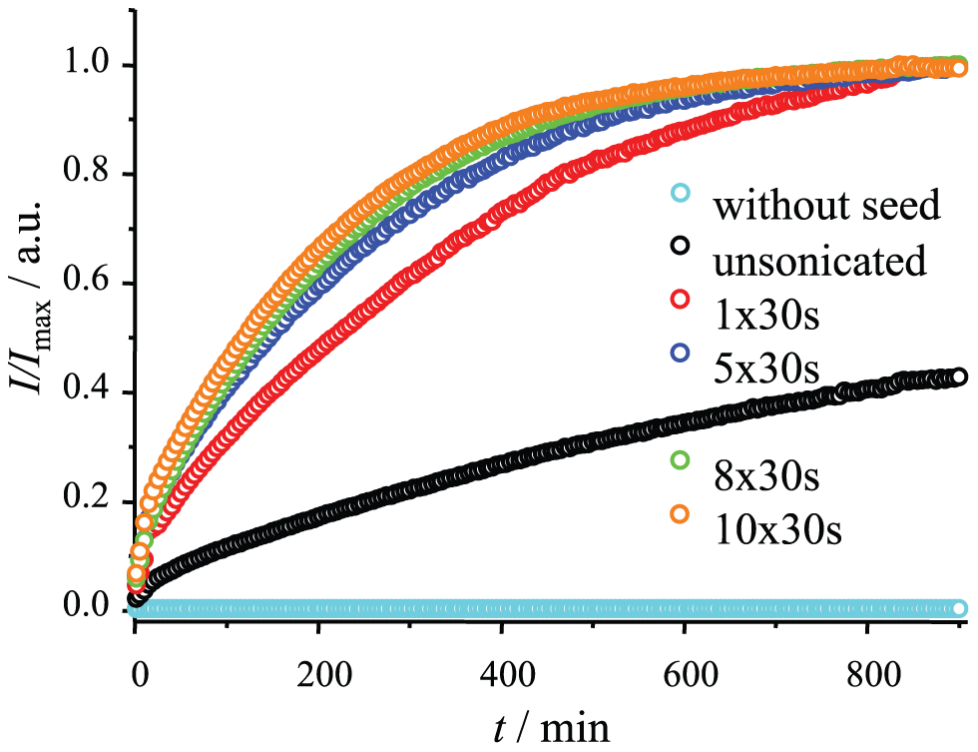
Different numbers of 30 second pulses were applied to fibrils before using them as seeds. Kinetics of elongation was followed by Thioflavin T assay at 37°C in the presence of 2 M guanidine hydrochloride.

Amyloid-like fibril formation includes several events – primary nucleation (*de novo* formation of amyloid-like particles), elongation (growth of fibrils via addition of new protein molecules) and secondary nucleation (increase of active fibril ends by breaking or branching of fibrils). Elongation is the main driving force for self-replication of amyloid-like fibrils. It can be initiated by adding preformed fibrils to a protein solution. Recombinant PrP can aggregate into fibrils under different conditions [3], [4], [14], [16], [17], but fibrils prepared by agitation at 37°C in presence of 2 M guanidine hydrochloride (GuHCl) [9], [14], [15] have been best characterized. Such fibrils were used as seeding material in our study. Ultrasonic treatment of fibrils is widely used in studies of amyloids and prions [18]–[22], and is known to break fibrils into shorter pieces [21], [22]. It helps both to homogenize fibril suspension, and to increase the rate of the process. Figure 1 shows a comparison of truncated mouse prion protein fibril elongation kinetics using seeds prepared using different sonication times. It is clear that sonication leads to several fold faster elongation rates, but every additional pulse is less efficient. Prolonged sonication may lead to minimum-sized amyloid-like fibrils [21], [23], and such fibrils seem to be the most efficient seeding material. Using sonicated fibrils as seeds under quiescent conditions gives the possibility of observing elongation without any visible nucleation events (Figure 1).

**Figure 2:**
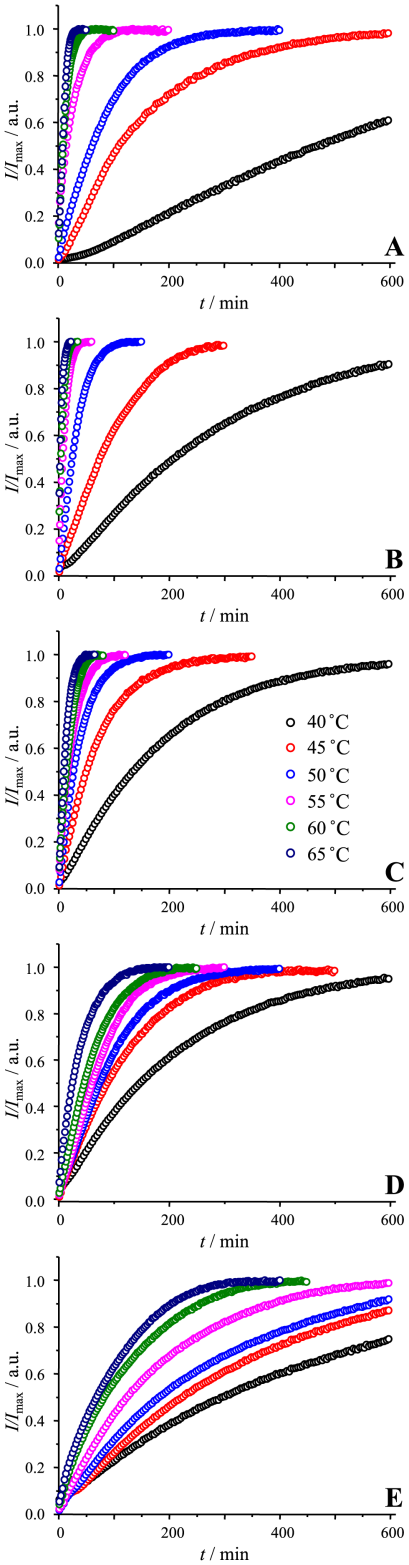
Fibril elongation at different temperatures in the presence of 0.5 M (A), 1 M (B), 1.5 M (C), 2 M (D) and 2.5 M (E) GuHCl.

**Figure 3:**
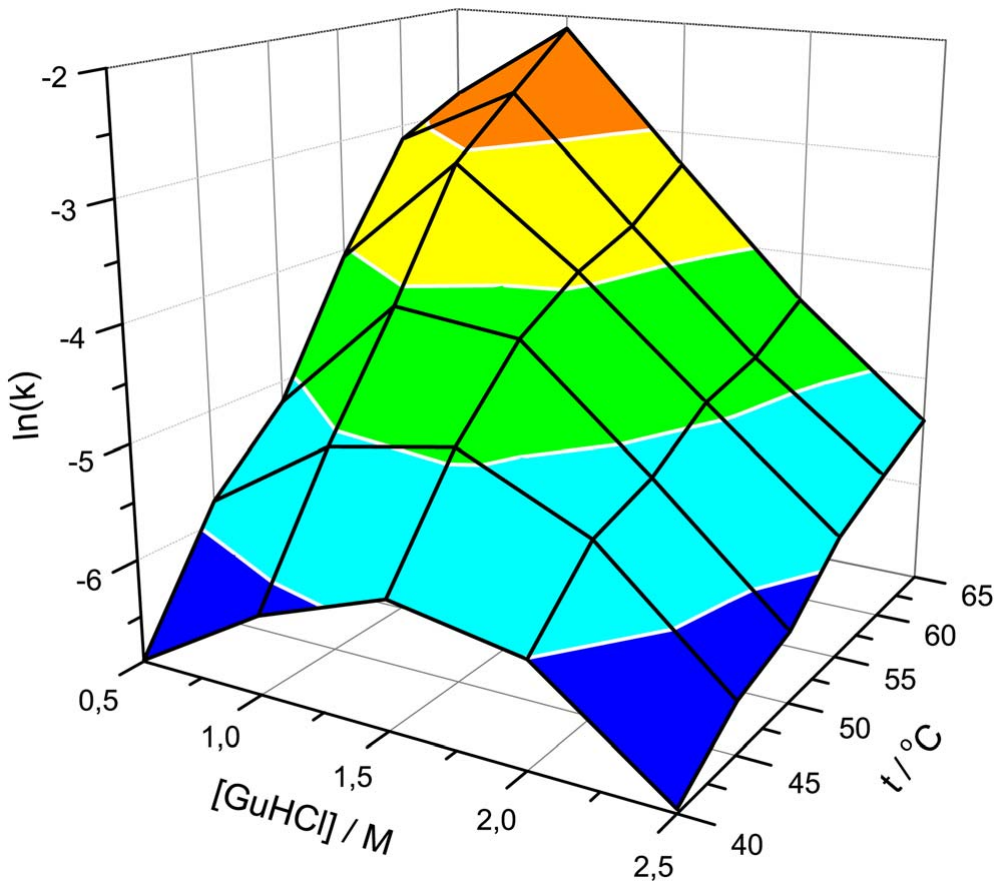
Three-dimensional landscape of fibril elongation rates at different temperatures and denaturant concentrations.

We followed elongation kinetics at six different temperatures in the presence of five different GuHCl concentrations (Figure 2). In all cases higher temperature leads to increased rates of elongation, however, the rate dependence on GuHCl concentration is more complicated. We summarized all measured elongation rates in Figure 3. At lower temperatures (40°C), the highest elongation rates are at moderate denaturant concentration (similar to previously reported moderate denaturant concentrations leading to the shortest lag times in spontaneous PrP fibrillation [24]), however, raising the temperature results in a decrease in optimal GuHCl concentrations.

**Figure 4:**
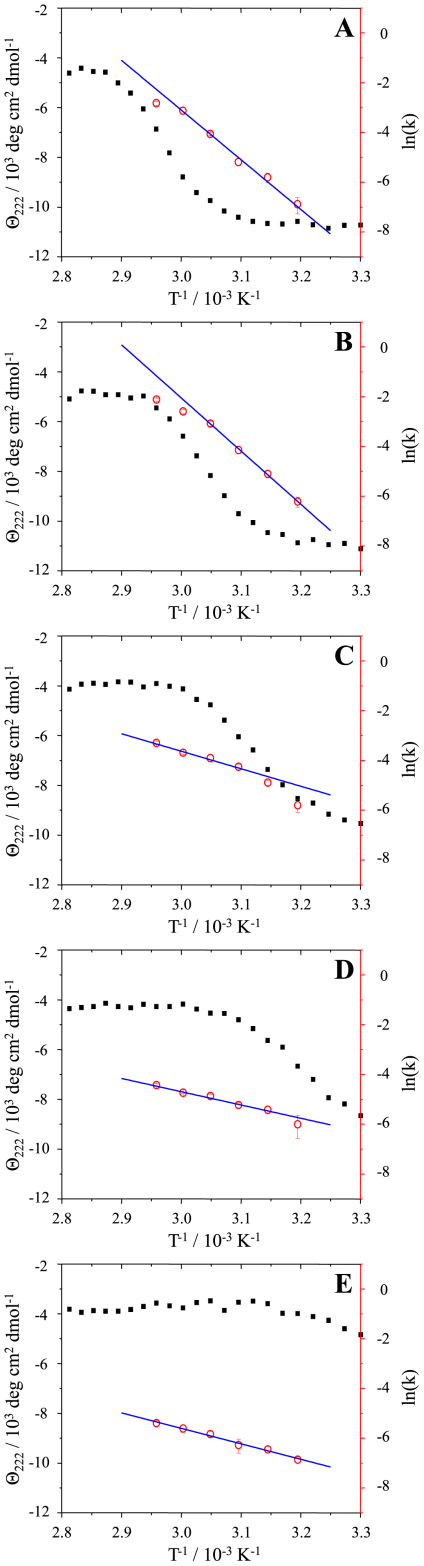
Comparison of Arrhenius plots with protein thermal unfolding curves in the presence of 0.5 M (A), 1 M (B), 1.5 M (C), 2 M (D) and 2.5 M (E) GuHCl. Black squares represent mean residue molar ellipticities, measured by circular dichroism, red circles represent the natural logarithm of elongation rates, and blue lines are the fits used to extract activation energies.

To understand the impact of denaturant, we plotted our kinetic data in an Arrhenius plot and compared them with MoPrP89-230 thermal denaturation curves, obtained from circular dichroism spectra (Figure 4). In the case of 0.5 M GuHCl (Figure 4A), the Arrhenius plot is linear over the range of 40–60°C (with calculated activation energy Ea = 165.8±10.0 kJ/mol); at this temperature range more than half of the protein is still folded. At higher temperature, most of the protein is unfolded and the rate of elongation does not follow the same line in the Arrhenius plot. Similar data can be observed for 1 M GuHCl (Figure 4B), but under such conditions, MoPrP89-230 unfolds at a lower temperature, thus, a valid linear fit for the Arrhenius plot is available only for the temperature range 40–55°C (Ea = 177.5±3.3 kJ/mol). At higher GuHCl concentrations most of the data were obtained at temperatures leading to a major fraction of unfolded protein, thus, a linear fit to the Arrhenius plot of fibril elongation at 1.5 M GuHCl (Figure 4C) is good for the temperature range 50–65°C (Ea = 58.3±6.3 kJ/mol), at 2 M GuHCl (Figure 4D) for 45–65°C (Ea = 44.4±4.9 kJ/mol), and at 2.5 M GuHCl (Figure 4E) for 40–65°C (Ea = 51.4±2.6 kJ/mol). Based on the data, two processes with different activation energies can be separated. Fibril elongation which assimilates folded PrP has greater energetic barrier (∼170 kJ/mol) than elongation using unfolded PrP (∼50 kJ/mol). Knowing that fibril formation requires complete rearrangement of native PrP secondary structure [9], [14], [15], it is easy to imagine that loss of secondary structure may significantly lower energetic barriers to the reaction.

**Figure 5:**
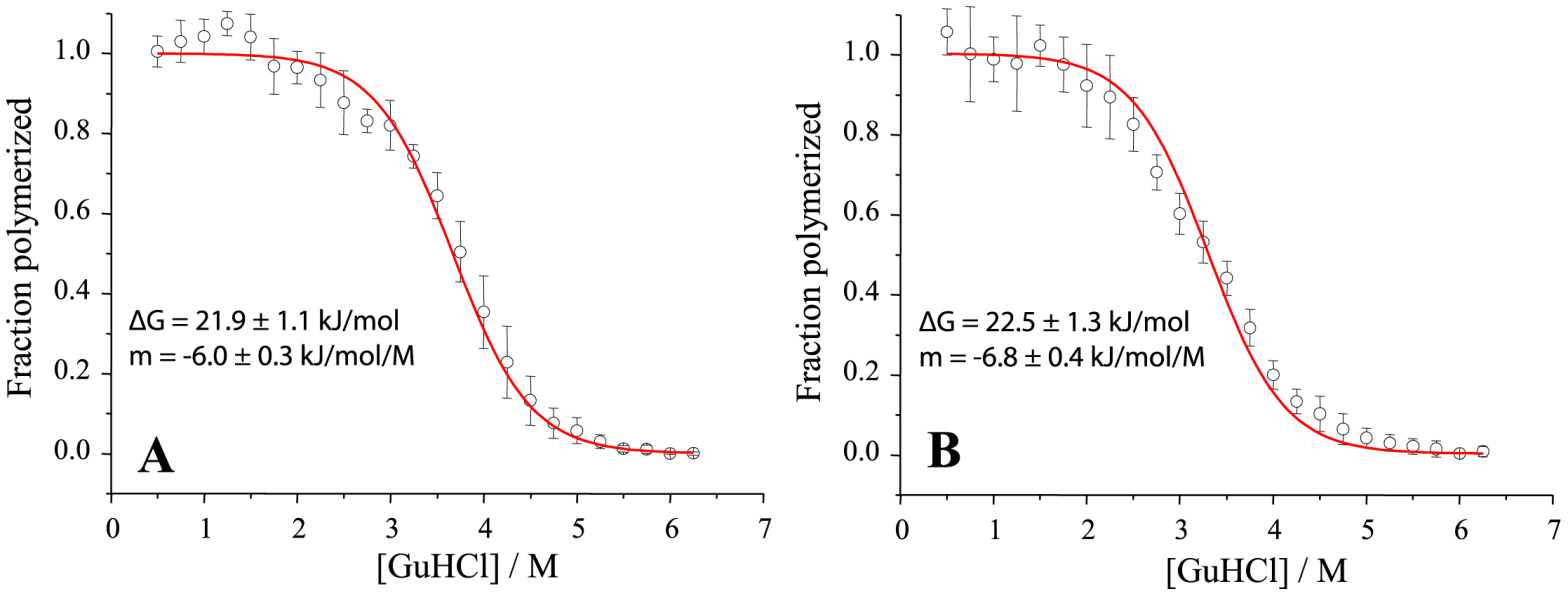
Denaturation profiles of MoPrP amyloid-like fibrils at ambient temperature (A) and at 60°C (B). These data were fitted to the two state depolymerization model.

To check monomer-fibril equilibrium dependence on temperature and denaturant we performed a fibril denaturation assay [25]. At ambient temperature (Figure 5A) fibrils are a bit more stable compared to fibers at 60°C (Figure 5B). It is important to note that even at high temperature fibrils are relatively stable up to 2 M GuHCl concentration (>96% of ThT fluorescence is retained). Thus it may only slightly impact (within one standard deviation) the estimated rate of elongation and activation energies. At 2.5 M GuHCl equilibrium shifts towards depolymerization of fibrils (∼94% ThT fluorescence retained at ambient temperature and ∼88% at 60°C). As we normalized all curves to the same final level, it should lead to overestimation of elongation rate. However, at the same time some of fibrils could be completely depolymerized, leading to lower numbers of fibril ends and thus underestimation of the rate. We can't quantify effects of both possible events, thus we state that errors of our measurements at 2.5 M GuHCl may reach up to 12%. This error does not affect our findings.

Buell and coworkers [26] compared elongation enthalpies for a number of amyloidogenic proteins and peptides and found that the presence of tertiary structure generally increases the enthalpy of activation per residue [26]. One of the facts supporting this finding is the difference in fibril elongation activation enthalpies between native and reduced forms of human lysozyme [26]. Interestingly, MoPrP89-230 and human lysozyme have similar numbers of amino acids, similar amounts of alpha-helices (∼40%) and comparable amounts of beta-sheets (<10%). Further, activation enthalpy for native human lysozyme (167.7±14.7 kJ/mol) [26] is very similar to activation energy for folded MoPrP89-230. However, activation enthalpy for reduced human lysozyme (68±12 kJ/mol) [26] is greater than activation energy for unfolded MoPrP89-230. Reduced lysozyme still has some secondary structure, which may be the reason for this difference.

Recently the idea of the prion-like nature of amyloid fibrils is widely discussed and gets some support from experimental data [27]–[30]. At this point, understanding fibril elongation as one of the possible driving forces of infectivity is a very important goal. Surprisingly, thermodynamics of fibril elongation has not yet attracted much attention. Most of the available data on this topic is summarized in the work of Buell and co-workers [26]. We expanded current knowledge by describing elongation of mammalian prion fibrils under a variety of conditions, including both folded and unfolded protein states. In fact, we demonstrated a possible way to separate the energies of monomer unfolding and refolding into an amyloid structure. Our findings may help in better understanding the fibril growth process and relations between prions and amyloids.

## Materials and Methods

Truncated mouse prion protein (MoPrP89-230) was expressed in *E. coli* and purified according to the previously described protocol [3]. After purification, the protein was stored frozen in 10 mM sodium acetate buffer, pH 4. In addition to the MoPrP sequence, the protein contains a 4-residue N-terminal extension (GSDP). Protein grade GuHCl (>99.7%) was obtained from Carl Roth. Thioflavin T (ThT) was obtained from Sigma. Sodium phosphates were obtained from Fisher Scientific UK.

To prepare fibrils, monomeric protein from a stock solution was diluted to a concentration of 0.5 mg/ml in 50 mM phosphate buffer (pH 6) containing 2 M GuHCl, and incubated for 3 days at 37°C with 220 rpm shaking (shaker incubator IKA KS 4000i). For seeding experiments fibrils were treated for 10 minutes using Bandelin Sonopuls 3100 ultrasonic homogenizer equipped with MS72 tip (using 20% power, cycles of 30 s/30 s sonication/rest, total energy applied to the sample per cycle ∼0.36 kJ). The sample was kept on ice during the sonication. Right after the treatment, 1 part fibrils was mixed with 19 parts 0.5 mg/ml of mouse prion solution containing 50 µM ThT and different concentrations of GuHCl in 50 mM phosphate buffer, pH 6. Elongation kinetics at different temperatures (40°C, 45°C, 50°C, 55°C, 60°C, 65°C) was monitored by ThT fluorescence assay (excitation at 470 nm, emission at 510 nm) using Qiagen Rotor-Gene Q real-time analyzer (see File S1 for broader description). ThT fluorescence curves were normalized by dividing each point by the maximum intensity of the curve. Rates of elongation were determined by linear fit of these curves in a range between 40–60% of the ordinate maxima (see File S1 for exponential fit comparison). Standard errors from 6 samples were calculated using Student's t-distribution at p = 0.05.

Thermal unfolding transition curves were measured on a Jasco J-815 circular dichroism spectropolarimeter For each experiment a sample of 0.05 mg/ml PrP was prepared in 50 mM phosphate buffer (pH 6) containing different amounts of GuHCl, and transferred to a 2 mm path length quartz cuvette. Ten CD spectra (in range 221–223 nm) were averaged for each temperature point. Temperature was raised by 2.5°C increments with an average rate of 1°C/min.

For chemical denaturation assays, amyloid fibrils were resuspended to a concentration of 25 µM in 50 mM phosphate buffer, pH 6, containing 0.5 M GuHCl and homogenized by sonication. These solutions were diluted 1∶4 in a buffer containing varying concentrations of GuHCl, and incubated for 60 min at 25°C or 60°C. Samples were then mixed 1∶20 with 50 µM ThT. Immediately after mixing, ThT fluorescence was measured at 480 nm using the excitation wavelength of 440 nm. The data was fit using a two state depolymerization model (each protein molecule would be either in fibrillar state and contribute to ThT fluorescence, or in monomer state). In this case the equilibrium constant is
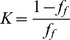
(1)where *f_f_* is fraction of protein molecules in fibrillar state. Assuming that the free energy of depolymerization (Δ*G_d_*) has a linear dependence on the concentration of the denaturant [31]

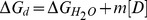
(2)and using its relation to equilibrium constant
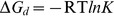
(3)it is possible to get the dependence of the fraction of fibrils on the concentration of GuHCl
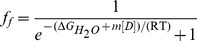
(4)


## Supporting Information

File S1 (PDF)
