# Supplementary material for: Elongation of Mouse Prion Protein Amyloid-Like Fibrils: Effect of Temperature and Denaturant Concentration
Source: PLoS One. 2014 Apr 18;9(4):e94469. doi: 10.1371/journal.pone.0094469 (PMC3991587; doi:10.1371/journal.pone.0094469)
Supplement: File S1 — (PDF) [file pone.0094469.s001.pdf]

Raw data illustration

Quiagen Rotor-Gene Q real-time analyzer is a PCR machine, which is designed for monitoring fluorescence of small volume samples at elevated temperatures. It has a channel for 470 nm excitation and 510 nm emission, which is not on the top of ThT excitation and emission peaks, however, the fluorescence intensity is still high enough for monitoring fibril formation. During the measurement, tubes are held in the rotor, which constantly rotates at 400 rpm. This helps to keep constant liquid volume at the bottom of the tube at elevated temperatures and still have the experiment at quiescent conditions. Unfortunately, part of the sample is out of reach of the beam, thus, absolute ThT intensity may differ from tube to tube (Figure 1A). But measurement of kinetics is almost unaffected (Figure 2B).

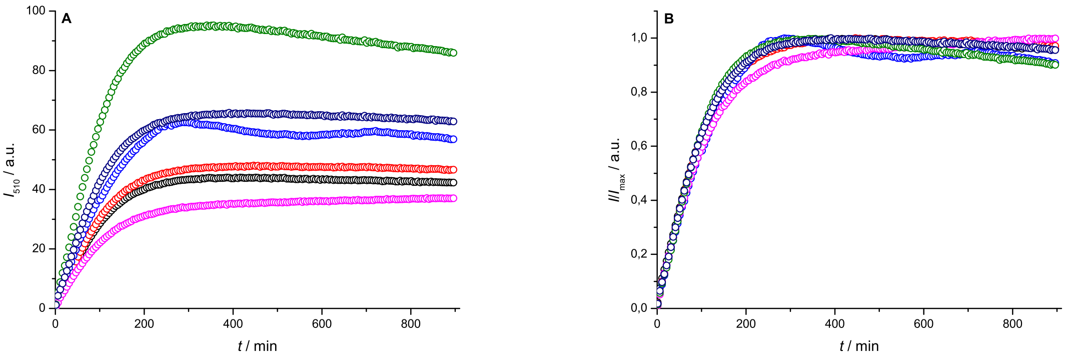

Figure 1. Elongation of MoPrP89-230 fibrils at 50°C (six repeats of the same batch sample). Raw (A) and normalized (B) curves.

Fitting

As suggested by reviewers, we also used fitted our data using exponential function

$$f(t) = 1 - Ae^{(-kt)} \tag{1}$$

where k is rate constant and A is a correction factor for initial fluorescence of added seeds. Temperature dependencies were similar as in case of using linear fit to obtain k values (Table 1).

Table 1.

	Activation energy [kJ/mol]	
C_{GuHCl} / [M]	linear fit[a]	exponential fit[a]
0.5	165.8 ± 10.0	162.0 ± 5.5
1	177.5 ± 3.3	184.1 ± 7.6
1.5	58.3 ± 6.3	62.7 ± 3.8
2	44.4 ± 4.9	48.8 ± 3.5
2.5	51.4 ± 2.6	56.0 ± 4.0

[a] Method, used for determination of rate constant.
